# Topological Vulnerability Evaluation Model Based on Fractal Dimension of Complex Networks

**DOI:** 10.1371/journal.pone.0146896

**Published:** 2016-01-11

**Authors:** Li Gou, Bo Wei, Rehan Sadiq, Yong Sadiq, Yong Deng

**Affiliations:** 1 School of Computer and Information Science, Southwest University, Chongqing 400715, China; 2 Department of Computer Science, Michigan Technological University, Houghton, MI 49931, United States of America; 3 School of Engineering, University of British Columbia Okanagan, 3333 University Way, Kelowna, BC, Canada V1V 1V7; 4 School of Engineering, Vanderbilt University, Nashville, TN 37235, United States of America; Kyushu University, JAPAN

## Abstract

With an increasing emphasis on network security, much more attentions have been attracted to the vulnerability of complex networks. In this paper, the fractal dimension, which can reflect space-filling capacity of networks, is redefined as the origin moment of the edge betweenness to obtain a more reasonable evaluation of vulnerability. The proposed model combining multiple evaluation indexes not only overcomes the shortage of average edge betweenness’s failing to evaluate vulnerability of some special networks, but also characterizes the topological structure and highlights the space-filling capacity of networks. The applications to six US airline networks illustrate the practicality and effectiveness of our proposed method, and the comparisons with three other commonly used methods further validate the superiority of our proposed method.

## 1 Introduction

Complex network is widely used to model the structure of many complex systems in nature and society [1, 2]. Some network models are used to solve natural problems, such as climate issues [3]. While some are used to analyze social and practical problems, such as supply chain management [4], transportation networks [5], power grid networks [6–9], water distribution networks [10], network optimization [11–13], as well as game theory in operation research [14, 15] and etc. An open issue is how to assess the vulnerability of complex networks [16–18], whose main objective is to understand, predict, and even control the behavior of a networked system under vicious attacks or any types of dysfunctions [19].

Different approaches to characterize network vulnerability and robustness have recently been proposed, which can be grouped into two types broadly [9, 20, 21]. The first type of the approaches is structural robustness—topological properties of networks [22, 23]. Such as, the average edge betweenness [19], the network connectivity level, the size of largest component [16] and the average geodesic length [6] and etc., are directly used to define network vulnerability. The second one concerns dynamical robustness [24–26]. The dysfunctions of a node or link will cause the redistribution on the the load of other nodes or links, with the risk that some other nodes or links may be overloaded, which will further cause a sequence of failures and even threaten the global stability [6, 20, 27]. Such behavior is called cascading failures [28–30]. In addition, the evaluation of network vulnerability is of uncertainty, and many methods have promising aspect to address such problem. For example, fuzzy set theory is efficient to model linguistic information [31] wihle Dempster-Shafer evidence theory can combine different information in an efficient manner [32–34]. The vulnerability analysis of complex systems, such as physic protect systems, is modelled by these mathematical tools [35].

One of the mostly used methods is proposed by Boccaletti *et.al* [19]. They construct a multi-scale evaluation model of vulnerability, which makes use of the average edge betweenness and introduces a key coefficient *p*. Due to its effectiveness, this method has been heavily studied [20]. One limitation of the original average edge betweenness model is that it cannot differentiate two different networks in some situations. To solve this problem, a key coefficient *p* is introduced to improve the original model in their work. However, a straight problem is how to determine the coefficient *p*. The way used in Boccaletti *et.al*’s work is lack of physical significance.

The main motivation of our work is that we believe this coefficient *p* should be determined by the network itself but not just geometrically. To address this issue, we take the fractal dimension of complex network into consideration.

Dimension is one of the fundamental properties of complex networks characterizing not only its topological properties but also dynamic characteristics [36–38], which are two key aspects to determine network vulnerability. Analyses of a variety of real complex networks show that self-similar characteristic exists on all length scales, that is, many complex networks exhibit fractal properties [23, 39, 40]. Then researchers found that this self-similar characteristic can used to characterize dimension, i.e., fractal dimension, which gives a good definition to dimension of many geometric images and the network with fractal properties, such as Koch curve, Sierpinski triangle, the Coast of Britain, social networks, power grid networks, airline networks and so on [41–43]. In addition, fractal dimension has also been confirmed to be able to measure the space-filling capacity of a pattern reflecting the complexity of network directly [44], which has relationships with network vulnerability. Based on this idea, we propose that fractal dimension is a promising alternative to be the key coefficient in determining network vulnerability in this paper.

This paper is organized as follows. Section 2 introduces the preliminaries. Section 3 presents details of the proposed method and the steps about its application. Section 4 compares the proposed method with the existing methods listed in other papers by calculating the vulnerability of them. Finally, we summarize our results in Section 5.

## 2 Preliminaries

In this section, we introduce Boccaletti *et.al*’s vulnerability model[19] and three other commonly used methods of vulnerability evaluation [6, 16, 20]. In general, the complex networks can be represented by an undirected and unweighted graph *G* = (*V*, *E*), where *V* is the set of nodes and *E* is the set of edges. Each edge connects exactly one pair of nodes, and a vertex-pair can be connected by maximally one edge, i.e. loop is not allowed.

### 2.1 Multi-scale Evaluation of Vulnerability

In Boccaletti *et.al*’s work [19], the original method to evaluate the vulnerability is represented by the average edge betweenness, which is defined as:

$$\begin{array}{c} b_{1}\left(G\right)=\frac{1}{\left|E\right|}\sum_{l\in E}b_{l}, \end{array}$$ (1)

Where |*E*| is the number of the edges, and *b*_*l*_ is the edge betweenness of the edge *l*, define as:

$$\begin{array}{c} b_{l}=\sum_{j,k\in V}\frac{n_{jk}\left(l\right)}{n_{jk}}. \end{array}$$ (2)

Where *n*_*jk*_ is the total number of geodesics (shortest path) from node *j* to *k*, and *n*_*jk*_(*l*) is the number of geodesics from *j* to *k* that contain the link *l*.

**Fig 1:**
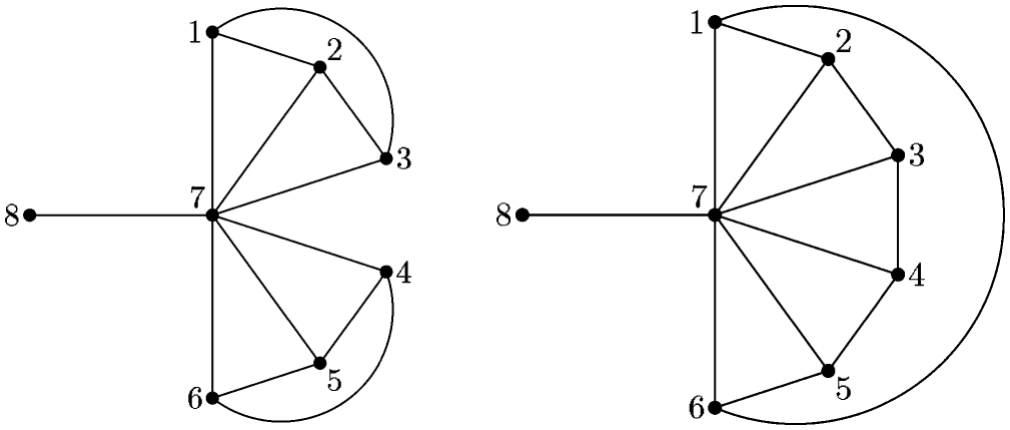
The “bat” graph *G* and the “umbrella” graph *G*′ [19].

However, this evaluation method of *b*_1_(*G*) gives no relevant new information about the vulnerability of some special networks. For example, two networks referred in [19] shown in Fig 1 can’t be distinguished using this method. By evaluating the vulnerability according to Eq 1, one gets *b*_1_(*G*) = *b*_1_(*G*′) = 43/13. It’s absolute that the “bat” graph *G* is more vulnerable than the “umbrella” graph *G*′, but Eq 1 gives the same evaluation results about them.

In order to overcome the original method’s limitation of failing to distinguish some networks, a key coefficient *p* was introduced by Boccaletti *et.al* in the improved model, which is called multi-scale evaluation of vulnerability [19] and shown as below:

$$\begin{array}{c} b_{p}\left(G\right)={\left(\frac{1}{\left|E\right|}\sum_{l\in E}b_{l}^{p}\right)}^{\frac{1}{\left|p\right|}}\;\left(p>0\right). \end{array}$$ (3)

If we want to compare two networks *G* and *G*′, first computes *b*_1_. If *b*_1_(*G*) < *b*_1_(*G*′), then *G* is more robust than *G*′. On the other hand, if *b*_1_(*G*) = *b*_1_(*G*′) then one takes *p* > 1 and computes *b*_*p*_ until *b*_*p*_(*G*) ≠ *b*_*p*_(*G*′).

To get the coefficient *p*, Boccaletti *et.al* define a relative function of *p* like:

$$\begin{array}{c} f\left(p\right)=\frac{|b_{p}\left(G\right)-b_{p}\left(G^{{}^{\prime }}\right)|}{\max\left(b_{p}\left(G\right),b_{p}\left(G^{{}^{\prime }}\right)\right)}. \end{array}$$ (4)

The coefficient *p* is obtained when the function has a maximal value. For more detailed information to determine the coefficient *p*, refer [19]. It’s clear that, the definition of coefficient *p* is based on geometrical definition and lack of physical significance. In our opinion, the coefficient *p* should be defined by the complex network itself, we take the fractal dimension as consideration.

### 2.2 Fractal Dimension

**Fig 2:**
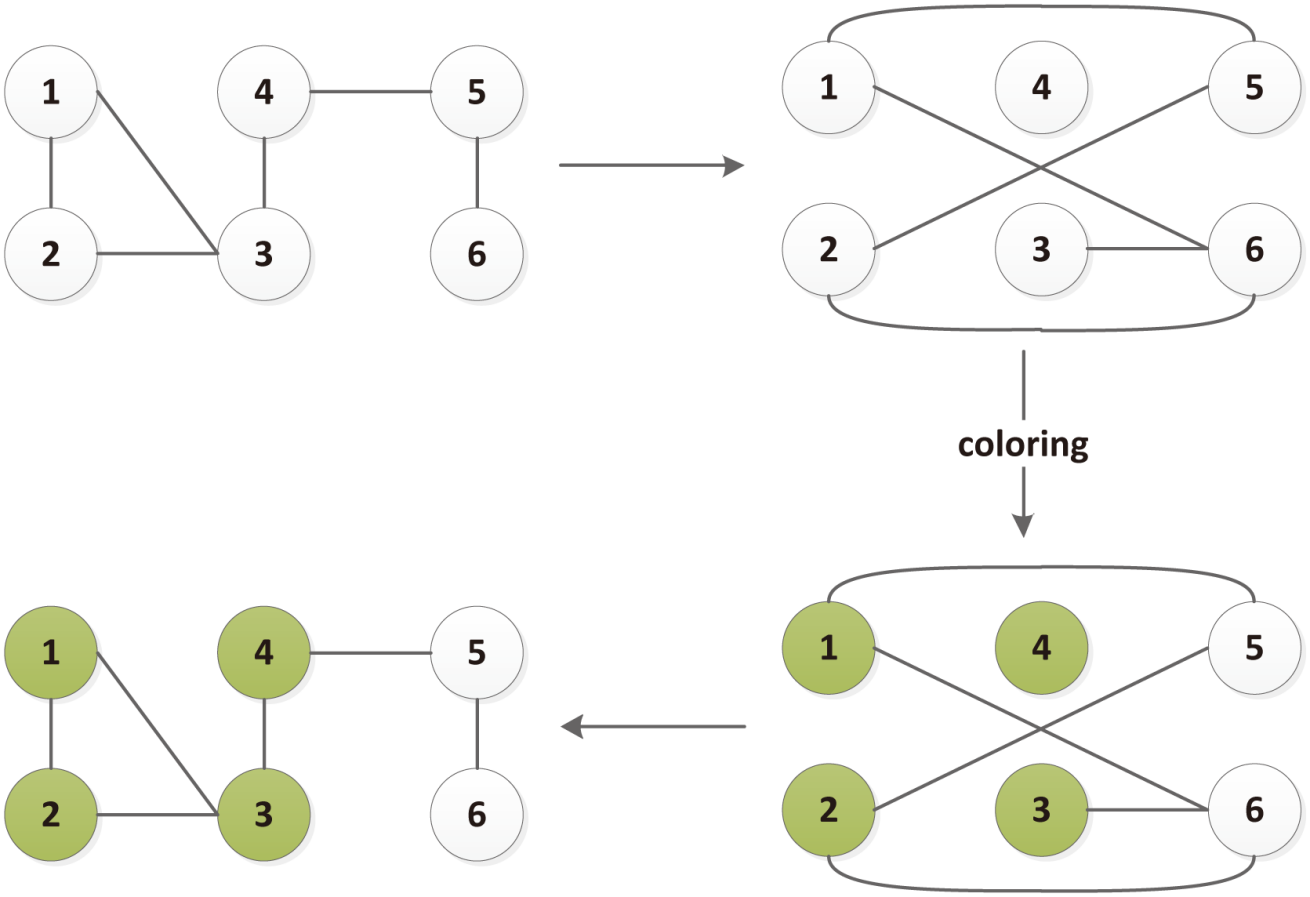
Illustration of the box-covering algorithms. Starting from *G* (upper left panel), a dual network *G*′ (upper right panel) was constructed for a given box size (here *l*_*B*_ = 3), where two nodes are connected if they are at a distance of *l* ≥ *l*_*B*_. A greedy algorithm was used for node colouring in *G*′, which is then used to determine the box covering in *G*, as shown in the plot [46].

One of the most typical ways to calculate the fractal dimension is Box covering algorithm, a power-law relation between the number of boxes needed to cover the network and the size of the box [45–47]. For a given network *G* and box size *l*_*B*_, a box is a set of nodes where all distances *l*_*ij*_ between any two nodes *i* and *j* in the box are smaller than *l*_*B*_. The minimum number of boxes required to cover the entire network is denoted by *N*_*B*_. The detailed illustration of the calculation of the fractal dimension referred in [46] is given in Fig 2. The fractal dimension or box dimension *d*_*B*_ calculated with the box covering algorithm is given as follows [40, 46]:

$$\begin{array}{c} N_{B}\approx l_{B}^{-d_{B}}. \end{array}$$ (5)

### 2.3 Comparison Preparation

For the sake of comparison, three other commonly used methods to calculate vulnerability are described as follows. The first method is the average inverse geodesic length *l*^−1^ [6]:

$$\begin{array}{c} l^{-1}=\langle \frac{1}{d\left(v,w\right)}\rangle \equiv \frac{1}{N(N-1)}\sum \sum \frac{1}{d\left(v,w\right)}. \end{array}$$ (6)

Where *d*(*v*, *w*) is the length of the geodesic between *v* and *w* (*v*, *w* ∈ *V*), and the factor *N*(*N* − 1) is the number of pairs of nodes. The larger *l*^−1^ is, the better the network functions.

The second method is the size of largest component *LCS* (0 < *LCS* < 1) [16], which quantifies the number of nodes in the largest connected subgraph and is defined as follows:

$$\begin{array}{c} LCS=\frac{N_{s}}{N}. \end{array}$$ (7)

Where *N*_*s*_ is the size of the largest connected subgraph. The larger *LCS* is, the more robust the network is.

And the third method is the normalized average edge betweenness *b*_*nor*_(*G*) [20], which is on the base of the Eq 3 while *p* = 1 and is defined as:

$$\begin{array}{c} b_{nor}\left(G\right)=\frac{b_{1}\left(G\right)-b_{1}\left(G_{complete}\right)}{b_{1}\left(G_{path}\right)-b_{1}\left(G_{complete}\right)}=\frac{b_{1}\left(G\right)-1}{\frac{N(N+1)}{6}-1}. \end{array}$$ (8)

Where *G*_*complete*_ is a complete graph and *G*_*path*_ is a path graph. The larger *LCS* is, the more vulnerable the network is.

## 3 Methods

In this section, the proposed method is detailed. As mentioned in introduction section, average edge betweenness can’t highlight difference between some special networks, which is caused by the average process. Because for a network whose geodesic distribution concentrates strongly around a single link or node, the potential risk of the failure in this critical link/node will be hidden by the average process with the rest of minor links. For example, edge betweenness of some edges in “bat” graph are much higher than “umbrella” graph, which represents the “bat” graph is more vulnerable than “umbrella” graph, but the average process hides their differences and gives the same vulnerability of them. The improved multi-scale evaluation model is equivalent to the *p* origin moment of edge betweenness. Since *p* origin moment can highlight the difference between data to eliminate the effect of average process. But the problem is how to determine the key coefficient *p* to make effective and reasonable evaluation, which is the motivation of our work.

In our opinion, the fractal dimension of the complex network is a promising alternative to redefine the coefficient *p*. It is well-known that the fractal dimension can characterize the network structure and basic physical properties including space-filling capacity. For a given network with fixed number of nodes, the higher the space-filling capacity is, the more edges connecting the nodes in this network, and then the smaller of the diameter of the network, thus the less boxes will be required to cover the whole network, i.e., the smaller the fractal dimension is. So the fractal dimension decreases with the space-filling capacity of network. We also know that given fixed number of nodes of a network, the more edges, the more connected and robust of this network. That is, the fractal dimension has direct relationships with network vulnerability. So using the fractal dimension to redefine *p* can not only highlight difference between networks properly, but also ensure practical significance of *p*, which is more reasonable than geometric coefficient *p* in multi-scale model. Therefore, we use the fractal dimension to redefine *p*.

**Definition.** Given a set of edge betweenness *B* = {*b*_*l*_1__, *b*_*l*_2__, …, *b*_*l*_*n*__} and fractal dimension *d*_*B*_ of network *G*, the proposed network vulnerability model is defined as:

$$\begin{array}{c} V_{d_{B}}\left(G\right)={\left(\frac{1}{\left|E\right|}\sum_{l\in E}b_{l}^{d_{B}}\right)}^{\frac{1}{|d_{B}|}}. \end{array}$$ (9)

The larger the *V*_*d*_*B*__, the more vulnerable the network. Any network that exhibits fractal properties can be applied to this proposed method to evaluate its vulnerability.

**Fig 3:**
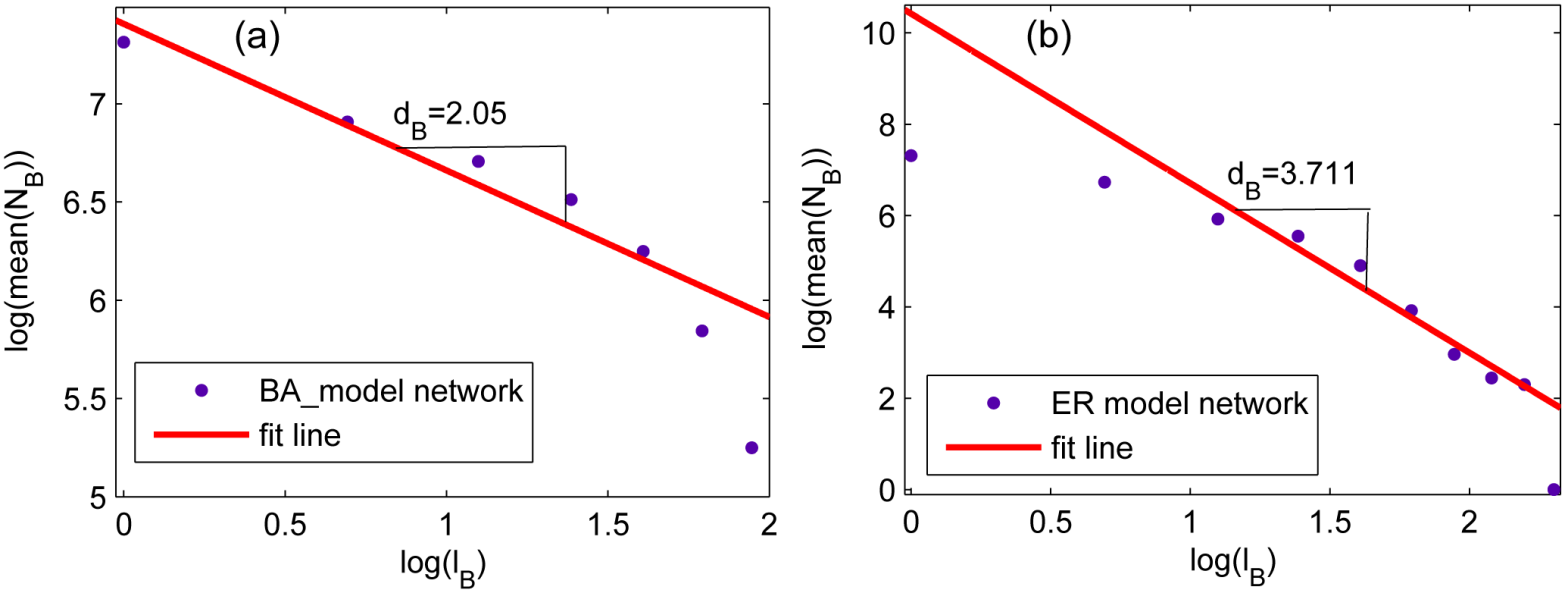
The *N*_*B*_ versus *l*_*B*_ of complex networks obtained in a log-log scale. (a) the ER network with the size *N* = 1500 and the average degree <*k*> = 6. (b) the BA network with *N* = 1500 and the average degree <*k* > = 4.8. The vertical ordinate of every subplot is the mean value of *N*_*B*_ for 100 times, and the horizonal ordinate represents the box size *l*_*B*_. The absolute value of the slope is the fractal dimension.

To demonstrate the usage of the proposed method, we apply it to two synthetic networks: Erdős-Rényi(ER) random networks [48] and Barabási-Albert(BA) model of scale-free networks [49]. The vulnerability of a network should be calculated as follow steps:

**Step 1** calculate the fractal dimension *d*_*B*_ of the network using box-covering algorithm [40, 46], i.e. Eq 5. The results of the two networks are illustrated in Fig 3.
**Step 2** Calculate the average edge betweenness according to Eq 2, and normalized by $\frac{N(N-1)}{2}$.
**Step 3** Calculate the vulnerability *V*_*d*_*B*__ in accordance with Eq 9.

Table 1 shows the results: *V*_*d*_*B*__(*BA*)>*V*_*d*_*B*__(*ER*), which mean that the ER network is more robust than the BA network. One point should be noted is that, the two networks are synthesized randomly, the results will vary with different network structure.

**Table 1:** General characteristics of the two networks: the number of nodes *N*, the average degree <*k*>, the fractal dimension *d*_*B*_, and the vulnerability *V*_*d*_*B*__ obtained by the proposed method.

network	*N*	<*k*>	*d*_*B*_	*V*_*d*_*B*__
ER	1500	6	3.711	0.0011
BA	1500	4.8	2.05	0.0014

## 4 Comparison and Discussion

In this section, to testify the correctness and effectiveness of the proposed method, three commonly used methods presented in section 2, that is, the average inverse geodesic length *l*^−1^, the largest component size *LCS* and the normalized average edge betweenness *b*_*nor*_(*G*), are applied to some real situations to compare with the proposed method. In order to get vulnerability embodying dynamic characteristics of networks to make a more reasonable comparison, we apply the RB attack strategy [6] to networks when using the three methods reflecting just static topological properties. RB attack strategy means that one should remove the node with highest betweenness value and recalculate the betweenness of the network. Similarly, removing other nodes until all required number of nodes have been removed. Finally, we can get the vulnerability of the network based on the remaining network. In this paper, *l*^−1^, *LCS* and *b*_*nor*_(*G*) are computed on attack strategy of removing 1% nodes from the original networks.

In this paper, six unweighed US airline networks, categorized by year, are used to do analysis. That is, US airline network in 2005, 2007, 2009, 2010, 2011, 2013 (UAN2005, UAN2007 and etc.). These data are downloaded from the Bureau of Transportation Statistics (BTS) Transtats site with the following filters [50]: Geography = all; Year = 2011; Months = all; and columns: Passengers, Origin, Dest. Based on this table, the airport codes are converted into id numbers; if there are flights between two airports, an edge between two nodes will be connected correspondingly. Also ties with a weight of 0 are removed (only cargo), self-loops and small subgraph are removed, constructing connected and unweighed networks.

**Fig 4:**
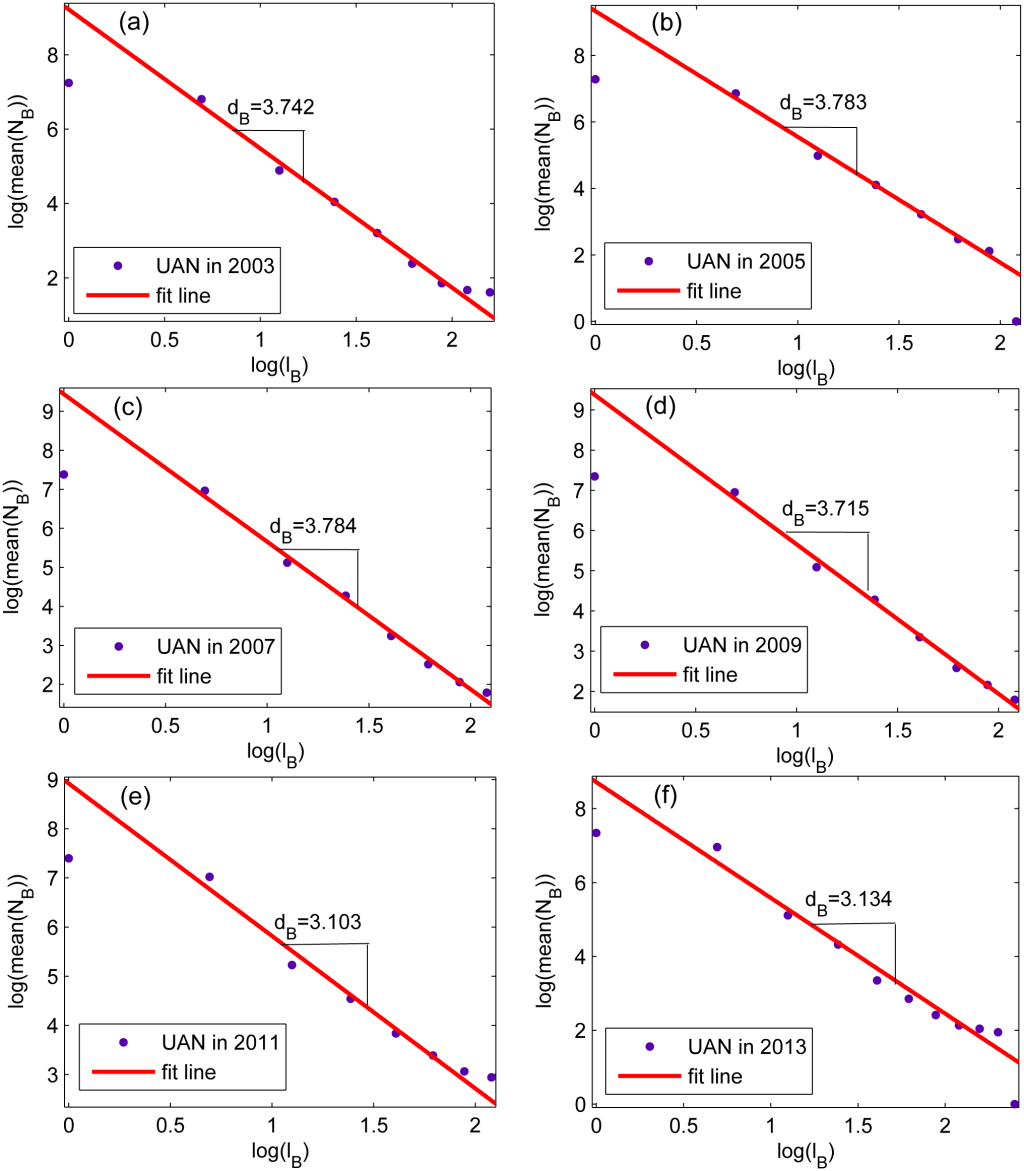
The *N*_*B*_ versus *l*_*B*_ of six real complex networks obtained in a log-log scale. the US airline network in 2003, 2005, 2007, 2009, 2011, 2013. The vertical ordinate of every subplot is the mean value of *N*_*B*_ (number of box required) for 100 times, and the horizonal ordinate represents the box size *l*_*B*_. The absolute value of the slope is the fractal dimension.

Firstly, *l*^−1^, *LCS* and *b*_*nor*_(*G*) are applied to these six airline network to calculate vulnerability, and the results are illustrated in Table 2. Then the fractal dimension of these networks are calculated and illustrated in Fig 4. It’s absolute that all these networks exhibit fractal properties, which reaches the prerequisite of the proposed method to use fractal dimension to characterize network vulnerability. Table 2 and Table 3 show the evaluation results of all these method, which are concluded as follows:

1. The average edge betweenness of these networks are very small, while the evaluation results of our method is larger than *b*_1_ at magnitude level, which indicates that the fractal dimension is appropriate to be the *p* origin moment of edge betweenness to highlight the difference between networks effectively.
2. The vulnerability order given by other three commonly used methods are unreasonable to some extent. *l*^−1^ and *LCS* show that UAN2013 is the most vulnerable network and *b*_*nor*_(*G*) illustrates a high robustness of UAN2003 and UAN2005.
3. Our method give the most reasonable results among these methods, and the vulnerability order obtained by it is: *UAN*2003 > *UAN*2005 > *UAN*2007 > *UAN*2009 > *UAN*2011 > *UAN*2013. The robustness of these airline networks increases over years, which is consistent with the real situations in the country.

**Table 2:** General characteristics of these networks and results illustrations. The proposed vulnerability evaluation method *V*_*d*_*B*__ is calculated based on fractal dimension *d*_*B*_. The normalized average inverse geodesic length $\tilde{l^{-1}}$, normalized largest component size $\tilde{LCS}$ and the normalized average edge betweenness *b*_*nor*_(*G*) are computed after 1% of vertices are removed and are normalized by the corresponding values of the initial networks.

network	N	E	*d*_*B*_	*V*_*d*_*B*__	*b*_1_	$\tilde{l^{-1}}$	$\tilde{LCS}$	*b*_*nor*_(*G*)
UAN2003	1387	15618	3.742	**0.0023**	0.00017	0.1221	0.3223	0.0096
UAN2005	1447	17453	3.783	**0.0021**	0.00018	0.1312	0.3034	-0.0984
UAN2007	1605	19166	3.784	**0.002**	0.00018	0.1389	0.3352	0.1051
UAN2009	1548	17415	3.715	**0.0019**	0.00020	0.1211	0.3204	0.0487
UAN2011	1587	17969	3.103	**0.0014**	0.00019	0.0916	0.2823	0.0961
UAN2013	1635	16215	3.134	**0.0013**	0.00021	0.0899	0.2557	0.042

**Table 3:** The vulnerability evaluation rank of all these methods.

Methods	vulnerability order
*V*_*d*_*B*__	*UAN*2003 > *UAN*2005 > *UAN*2007 > *UAN*2009 > *UAN*2011 > *UAN*2013
$\tilde{l^{-1}}$	*UAN*2013 > *UAN*2011 > *UAN*2009 > *UAN*2003 > *UAN*2005 > *UAN*2007
$\tilde{LCS}$	*UAN*2013 > *UAN*2011 > *UAN*2005 > *UAN*2009 > *UAN*2003 > *UAN*2007
*b*_*nor*_(*G*)	*UAN*2007 > *UAN*2011 > *UAN*2009 > *UAN*2013 > *UAN*2003 > *UAN*2005

The obvious advantage of the proposed method is due to its multiple evaluation index, that is, edge betweenness, *p* origin moment and fractal dimension, which are all crucial to the vulnerability evaluation as described in section 3. While the evaluation index of other three methods is obvious inferior to ours, in detail, $\tilde{l^{-1}}$ just utilizes the geodesic length of network, $\tilde{LCS}$ just considers the simple structure properties–connectivity, and *b*_*nor*_(*G*) only involves the average betweeness.

In conclusion, *p* origin moment is an effective approach to improve the average process of edge betweenness and the application of fractal dimension can highlight the difference between networks efficiently. So the proposed method has its geometrical and physical significance, and the reasonable application results also illustrate its effectiveness and practicality.

## 5 Conclusions

In this paper, the fractal dimension is redefined as p origin moment of edge betweenness to improve the vulnerability evaluation of multi-scale model. The experiments indicate that the fractal dimension indeed has relationships with network vulnerability, because a higher space-filling capacity means that less boxes will be required to cover the whole network, thus with smaller fractal dimension; while a network with higher space-filling capacity will contribute to more connected and robust structure. The applications to several real airline networks and comparison with other commonly used methods also illustrate the effectiveness and practicability of using the fractal dimension to evaluate network vulnerability. One of our ongoing works is to explore the specific relationship between vulnerability and topological properties, such as connectivity, cluster degrees and so on.
